# An Analytical Energy Harvester Model for Interdigitated Ring Electrode on Circular Elastic Membrane

**DOI:** 10.3390/mi13010133

**Published:** 2022-01-15

**Authors:** Hua-Ju Shih, Kuo-Ching Chen

**Affiliations:** Institute of Applied Mechanics, National Taiwan University, Taipei 10617, Taiwan; D04543001@ntu.edu.tw

**Keywords:** variable capacitance, interdigitated ring electrodes, vibration of circular membranes, conformal mapping

## Abstract

Energy harvesters are devices that accumulate ambient vibrational energy from the environment, and for the time being, variable capacitance is the most widely used mechanism. Various designs were proposed to increase the power of such devices, and in particular, the interdigitated electrode (IDE) pattern is the mainstream. Nevertheless, most IDE designs focus merely on the parallel-type vibrations of electrodes. In this study, the performance of a novel harvester, which combined circular membrane and interdigitated ring electrodes (IRE), was investigated. This design allows the device to collect energy from the rotational structure motions of electrodes through the vibrating membrane. Besides, the circular structure provides a dense capacitive arrangement that is higher than that of the arrangement obtained using regular rectangular chips. The IRE diagram is composed of many capacitive rings, each of which harvests vibrated energy simultaneously. Three gaps (1, 10, and 100 μm) of the ring are investigated for the first four vibrational modes of the membrane to understand the effect of energy output. It is found that the energy outputs are approximately the same for the three gaps; however, rings with a wider gap are easier to manufacture in MEMS.

## 1. Introduction

The mechanism of variable capacitance is widely used in vibration energy harvesters (VEHs), such as reverse electrowetting [1,2], honeycomb [3], electrostatic levitation [4], and interdigitated electrode (IDE) devices. According to the movement direction of the electrode plates, variable capacitance can be classified into three types [5], namely in-plane overlap [6], in-plane gap-closing [7], and out-of-plane gap-closing [8]. The in-plane overlap harvester varies the overlap area between finger electrodes, the in-plane gap-closing one varies the gap between finger electrodes, and the out-of-plane gap-closing one varies the gap between two large electrode plates. It is noted that these harvesters focus merely on the parallel-type vibrations of adjacent electrodes manufactured on rectangular chips.

Among various structures of a variable capacitance, the IDE design is commonly used in microelectromechanical systems [9,10]. This design possesses two advantages. First, the fabrication process is simple because the design of the IDE pattern can be produced in a plane structure. Second, a high-density arrangement of electrodes can be achieved on a micro scale. Different from the IDE but endowed with the same advantages, there is a radial geometric pattern of interdigitated ring electrode (IRE) that was developed by NASA to provide a radially distributed electric field in a circular piezoelectric actuator [11]. To date, the IRE design is widely used in biotechnology and flow sensors [12,13,14]; however, little attention has been paid to the study of this pattern applied to energy harvesting.

Here, a novel harvester with variable capacitance is proposed. The harvester is composed of a flexible circular membrane and a metal conductor with a certain IRE pattern. This device enables the energy collection from the vibration of radial electrodes on the diaphragm rather than the parallel motions of adjacent electrodes. Moreover, our design has two advantages. First, for the sake of resonance, we are able to tune the natural frequency of the device by adjusting the tension force of the membrane to match the vibration frequency from the environment. Secondly, the flexible structure of this device can sustain larger deformation from the external force than a harvester, which uses piezoelectric material.

Since our harvester has high-density ringed electrodes, a computational process would be time-consuming by using the finite element method (FEM). As a result, the conformal mapping method, which transforms the non-uniform electric field into a uniform one, is employed so that we are able to quickly evaluate the electric field of the IRE and thus estimate the capacitance variation per cycle of the harvester.

In this paper, we propose a mathematical model to analytically investigate the performance of the energy harvester with the IRE on a circular membrane. Since the membrane vibration gives rise to the angle change between two adjacent electrodes, the harvester achieves charge–discharge behavior. In order to evaluate the ability of harvesting energy due to angle change in the rotational electrodes, we quantitatively compared the capacitive change in a capacitor using rotating electrodes (applied to the circular membrane) with that using non-rotating electrodes (applied to the rectangular chip). Furthermore, the power density of our harvester is discussed in the case of a capacitive ring gap of 100 μm for 30 Hz vibration. Although this work is a theoretical discussion, our analysis provides useful information that pure experiments cannot offer. Such information includes the modal shape of the circular membrane and the angle profile of ringed electrodes on the deformed membrane, which enables us to find the optimal distribution of electrodes to raise the power of the harvester.

## 2. Mathematical Model

### 2.1. Vibration of Circular Membranes

**Figure 1:**
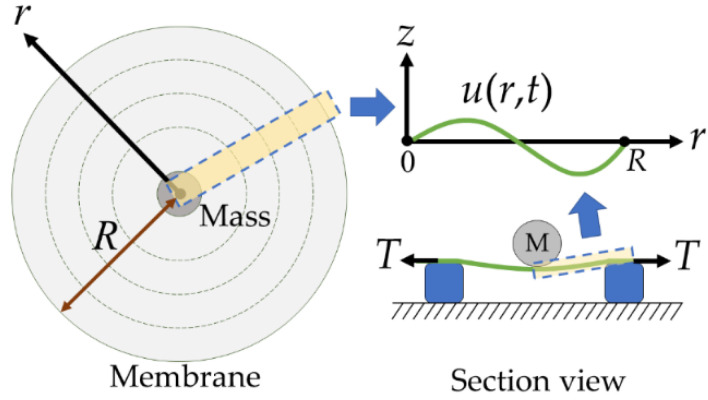
Top and section views of the circular membrane.

We assume that a circular and symmetrical membrane is made of flexible elastic, and the vibration of the membrane can be modeled by the wave equation. By considering this membrane of radius *R* in Figure 1, the governing equation of vibration in the polar coordinate system (*r, θ, z*) can be expressed as

(1) $$\begin{array}{l} \frac{\partial^{2}u}{\partial t^{2}}=c^{2}\left(\frac{\partial^{2}u}{\partial r^{2}}+\frac{1}{r}\frac{\partial u}{\partial r}\right),\\ u(R,t)=0\;\mathrm{for}\;\mathrm{all}\;t\geq 0,\\ u\left(r,0\right)=f\left(r\right),\\ \frac{\partial u}{\partial t}\left(r,0\right)=g\left(r\right), \end{array}$$

where *u*(*r, t*) is the displacement function of the membrane in the *z*-direction, which solely depends on the *r*-axis and time *t*. Here, *c* is the wave velocity, and *f*(*r*) and *g*(*r*) are the profiles of the initial deflection and initial velocity, respectively.

Equation (1) can be readily resolved using the separation of variables [15] as follows

(2) $$\begin{array}{l} u_{m}(r,t)=G_{m}(t)W_{m}(r)=\left(A_{m}\cos\lambda_{m}t+B_{m}\sin\lambda_{m}t\right)J_{0}\left(\frac{\alpha_{m}}{R}r\right),\\ A_{m}=\frac{2}{R^{2}J_{1}^{2}\left(\alpha_{m}\right)}\int_{0}^{R}rf(r)J_{0}\left(\frac{\alpha_{m}}{R}r\right)dr,\\ B_{m}=\frac{2}{\left(c\alpha_{m}R\right)J_{1}^{2}\left(\alpha_{m}\right)}\int_{0}^{R}rg(r)J_{0}\left(\frac{\alpha_{m}}{R}r\right)dr, \end{array}$$

where *λ_m_* is the eigenvalue for the *m*th mode, and it comprises the wave velocity and wave number (*λ_m_* = *ck_m_* = *cα_m_*/*R*). *W_m_*(*r*) is the eigenfunction; *J*_0_ and *J*_1_ are the zeroth- and first-order Bessel functions of the first kind, respectively, and *A_m_* and *B_m_* are the coefficients. The values of *α_m_* for the first four modes are listed in Table 1.

As depicted in Figure 1, the membrane, subjected to a pretension *T_p_* and a mass *M* on the circular center, is supposed to have a tension *T* along the *r* direction. The velocity of the wave *c* can be expressed as *c* = (*T*/*ρ*)^0.5^, with the density of the membrane *ρ*. For simplicity, the initial deflection curve of the membrane is simulated by

(3) $$f(r)=\frac{\delta }{2}\left[-1+\cos\pi \left(1-\frac{r}{R}\right)\right],$$

where *δ* is the subsidence of the membrane center point. We assume the initial velocity *g*(*r*) = 0. Therefore, Equation (2) is simply written as

(4) $$\begin{array}{ll} u_{m}(r,t) & =J_{0}\left(\frac{\alpha_{m}}{R}r\right)A_{m}\cos\left(\lambda_{m}t\right)\\ & =J_{0}\left(\frac{\alpha_{m}}{R}r\right)\cos\left(c\frac{\alpha_{m}}{R}t\right)\left[\frac{2}{R^{2}J_{1}^{2}\left(\alpha_{m}\right)}\int_{0}^{R}rf(r)J_{0}\left(\frac{\alpha_{m}}{R}r\right)dr\right]. \end{array}$$

We are able to obtain the vibration of the membrane *u_m_* in the *m*th normal mode by using Equation (4) as soon as *R*, *c*, and *δ* are specified.

### 2.2. Interdigitated Ring Electrode on the Circle Membrane

**Figure 2:**
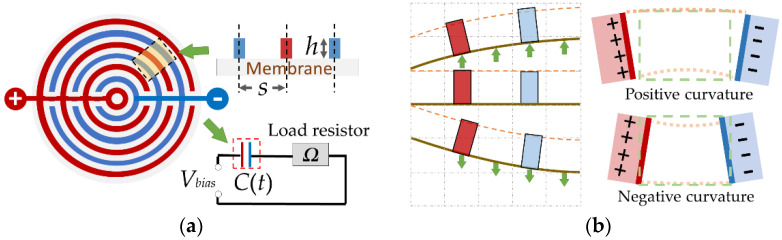
Schematic of the circular membrane energy harvester: (**a**) nomenclature of the circular membrane of variable capacitance C(*t*) and the equivalent circuit diagram; (**b**) mechanism of the circular membrane energy harvester.

The IRE on the circular membrane consists of two electrode lines (denoted by the red and blue lines in Figure 2a). When applying the voltage *V_bias_*, the loop, in Figure 2a, can be regarded as a resistor–capacitor equivalent circuit. The behavior of charge and discharge for the variable capacitor is from the capacitive change. That is why the IRE can harvest energy through membrane vibration.

Because the circular membrane is symmetrical, the deflection of the ring in the membrane is the same for any given radius *r* (independent on *θ*). Therefore, we assume that the circular membrane is cut into (*R*/*s* − 1) rings. Every ring contains a positive electrode and a negative electrode, thus forming a variable capacitor structure. The total length of the capacitance in a circular membrane is expressed as follows

(5) $$\begin{array}{ll} L_{C} & =\sum_{n=1}^{N}2\pi r_{n}\\ & =\sum_{n=1}^{N}2\pi \left(n+0.5\right)s, \end{array}$$

where *N* = (*R*/*s* − 1). The parameter *s* represents the gap of the capacitive ring, and *r_n_* is the *n*-th radius.

### 2.3. Electrostatic Capacitance

**Figure 3:**
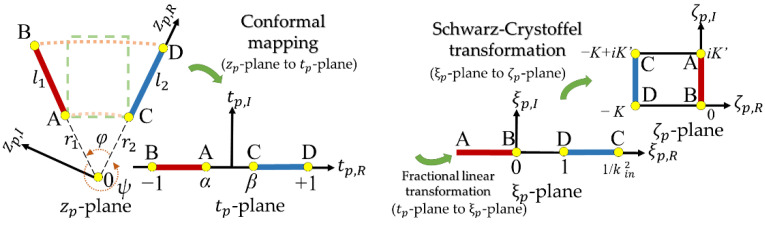
Conformal mapping transformation process for two electrodes per unit longitudinal length. First, the cross-section of the inclined-plate capacitor of the ring on the membrane (*z_p_*-plane) is mapped onto the upper half plane (*t_p_*-plane). Second, the *t_p_*-plane is mapped onto the *ξ_p_*-plane through fractional linear transformation. Third, the *ξ_p_*-plane is mapped onto the *ζ_p_* -plane through the Schwarz-Crystoffel transformation.

The variable capacitance in the ring results in the formation of an inclined-plate capacitor when the membrane vibrates (Figure 2b). In order to calculate the electrostatic capacitance precisely, the method described in [16] is used for calculating the electrostatic capacitance per unit longitudinal length of an inclined plate,

(6) $$\begin{array}{ll} C & =C_{in}+C_{out}\\ & =\varepsilon_{0}\frac{K\prime (k_{in})}{K(k_{in})}+\varepsilon_{0}\frac{K\prime (k_{out})}{K(k_{out})}\\ & =\varepsilon_{0}\frac{K(k_{in}\prime )}{K(k_{in})}+\varepsilon_{0}\frac{K(k_{out}\prime )}{K(k_{out})}, \end{array}$$

where *C_in_* is the internal capacitance inside angle *φ* per unit longitudinal length; *C_out_* is the external capacitance outside angle *ψ* per unit longitudinal length (Figure 3); *ε*_0_ is the permittivity of vacuum; *K*(*k*) is the complete elliptic integral of the first kind; and *k_in_*, *k′_in_*, *k_out_*, and *k′_out_* are the modulus. In addition, the method assumes the width of the electrode is negligible.

Figure 3 illustrates the conformal mapping transformation process for an inclined-plate capacitor per unit longitudinal length transforming to a parallel-plate capacitor configuration. In Table 2, three transfer equations were used to derive Equation (6) for the equivalent parallel-plate capacitor (*C_in_* and *C_out_*) in the *ζ_p_* -plane. We know that obtaining the inclined-plate capacitance is not an easy task, so using the conformal mapping method is more straightforward because it transforms the problem into a set of simpler algebra equations.

Equation (6) indicates that *C_in_* and *C_out_* are from an electrostatic field enclosed by the two electrodes in the interior of angles *φ* and *ψ*. The modulus *k_in_* is related to the inside angle *φ*

(7) $$k_{in}=\sqrt{\frac{\left(r_{1}^{\pi /\varphi }+r_{2}^{\pi /\varphi }\right)\left({\left(r_{1}+l_{1}\right)}^{\pi /\varphi }+{\left(r_{2}+l_{2}\right)}^{\pi /\varphi }\right)}{\left(r_{1}^{\pi /\varphi }+{\left(r_{2}+l_{2}\right)}^{\pi /\varphi }\right)\left(r_{2}^{\pi /\varphi }+{\left(r_{1}+l_{1}\right)}^{\pi /\varphi }\right)}},$$

and the complementary modulus of *k′_in_* is expressed as

(8) $$\begin{array}{ll} k_{in}^{\prime } & =\sqrt{1-k_{in}{}^{2}}\\ & =\sqrt{\frac{\left({\left(r_{1}+l_{1}\right)}^{\pi /\varphi }-r_{1}^{\pi /\varphi }\right)\left({\left(r_{2}+l_{2}\right)}^{\pi /\varphi }-r_{2}^{\pi /\varphi }\right)}{\left(r_{1}^{\pi /\varphi }+{\left(r_{2}+l_{2}\right)}^{\pi /\varphi }\right)\left(r_{2}^{\pi /\varphi }+{\left(r_{1}+l_{1}\right)}^{\pi /\varphi }\right)}}. \end{array}$$

Additionally, for the field region of angle ψ, we can determine *k_out_* and *k*′*_out_* by using the following expressions

(9) $$k_{out}=\sqrt{\frac{\left(r_{1}^{\pi /\psi }+r_{2}^{\pi /\psi }\right)\left({\left(r_{1}+l_{1}\right)}^{\pi /\psi }+{\left(r_{2}+l_{2}\right)}^{\pi /\psi }\right)}{\left(r_{1}^{\pi /\psi }+{\left(r_{2}+l_{2}\right)}^{\pi /\psi }\right)\left(r_{2}^{\pi /\psi }+{\left(r_{1}+l_{1}\right)}^{\pi /\psi }\right)}},$$

and

(10) $$k_{out}^{\prime }=\sqrt{\frac{\left({\left(r_{1}+l_{1}\right)}^{\pi /\psi }-r_{1}^{\pi /\psi }\right)\left({\left(r_{2}+l_{2}\right)}^{\pi /\psi }-r_{2}^{\pi /\psi }\right)}{\left(r_{1}^{\pi /\psi }+{\left(r_{2}+l_{2}\right)}^{\pi /\psi }\right)\left(r_{2}^{\pi /\psi }+{\left(r_{1}+l_{1}\right)}^{\pi /\psi }\right)}}.$$

**Figure 4:**
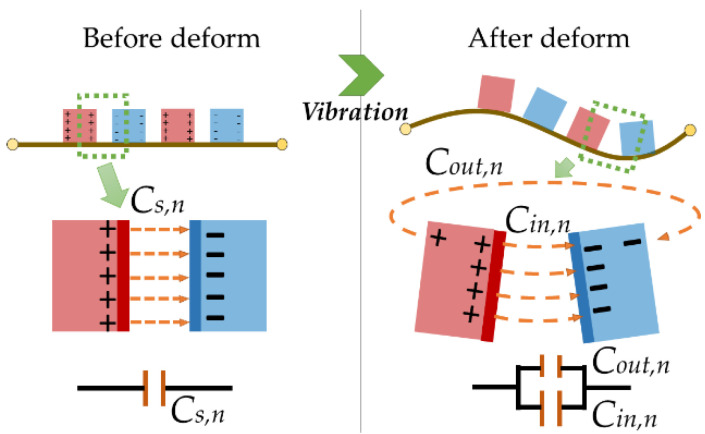
Capacitance on the circular membrane before and after vibration.

We consider that the surface charge of the electrodes on the membrane is redistributed after vibration (Figure 4) and do not consider the interaction between the capacitors. By using Equations (5) and (6), the capacitive variation in the *n*th electrode ring and the total capacitive variation can be calculated as follows

(11) $$\begin{array}{l} C_{d,n}=2\pi r_{n}\left(C_{n}-C_{s}\right)=2\pi r_{n}\left[\varepsilon_{0}\left(C_{in,n}+C_{out,n}\right)-\varepsilon_{0}\frac{h}{s}\right],\\ C_{d}=\sum_{n=1}^{N}\left(C_{d,n}\right), \end{array}$$

where *C_n_* is the *n*-th capacitance per unit longitudinal length, which is composed by the internal capacitance *C_in,n_* and the external capacitance *C _out,n_*; *C_s_* is the capacitance before vibration; and *C_d_* is the total capacitive variation, which is the sum of the capacitive variation in electrode rings on the membrane.

Based on the voltage-constrained conversion cycle [17] and Equations (4)–(6), the energy gain per vibration cycle of the harvester is expressed as follows

(12) $$\begin{array}{ll} E & =\left(0.5\times \Delta C\times V_{bias}^{2}\right)\\ & =\left(0.5\times 2\left|C_{d,\max}\right|\times V_{bias}^{2}\right)\\ & =2\varepsilon_{0}\pi V_{bias}^{2}\times \left|\sum_{n=1}^{N}s\left(n+0.5\right)\left[\left(\frac{K(k_{in,n}^{\prime })}{K(k_{in,n})}+\frac{K(k_{out,n}^{\prime })}{K(k_{out,n})}\right)-\frac{h}{s}\right]\right|, \end{array}$$

where Δ*C* is the capacitance difference, which is the difference of peak and valley capacitance. For our harvester, the capacitance depends on the periodic deflection of the membrane *u_m_*(*r*,*t*) with time, so that Δ*C* equals (2│*C_d_*_,max_│).

## 3. Results and Discussion

**Figure 5:**
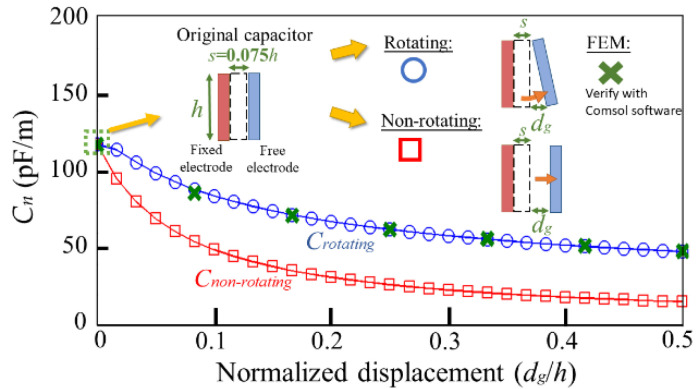
Capacitance per unit length versus normalized displacement for the rotating and non-rotating electrodes.

In order to examine the effect of the inclined-plate capacitor on the circular membrane, the rotating and the non-rotating electrode structures are discussed. A comparison of the capacitive change over the normalized displacement between the rotating structure vs. the non-rotating structure is provided in Figure 5, where the capacitance of the rotating structure is calculated using Equation (6), and the capacitance of the non-rotating structure is obtained from the ideal capacitance formula. In addition, we verified seven sets of data of the rotating structure (*d_g_*/*h* = 0, 0.09, 0.17, 0.26, 0.34, 0.42, 0.5) by FEM, and the relative errors of capacitance between using Equation (6) and FEM are less than 2.7%. It is seen from Figure 5 that the capacitive change in the non-rotating structure is greater than that of the rotating structure, meaning that the former is able to collect more energy with the same displacement.

**Figure 6:**
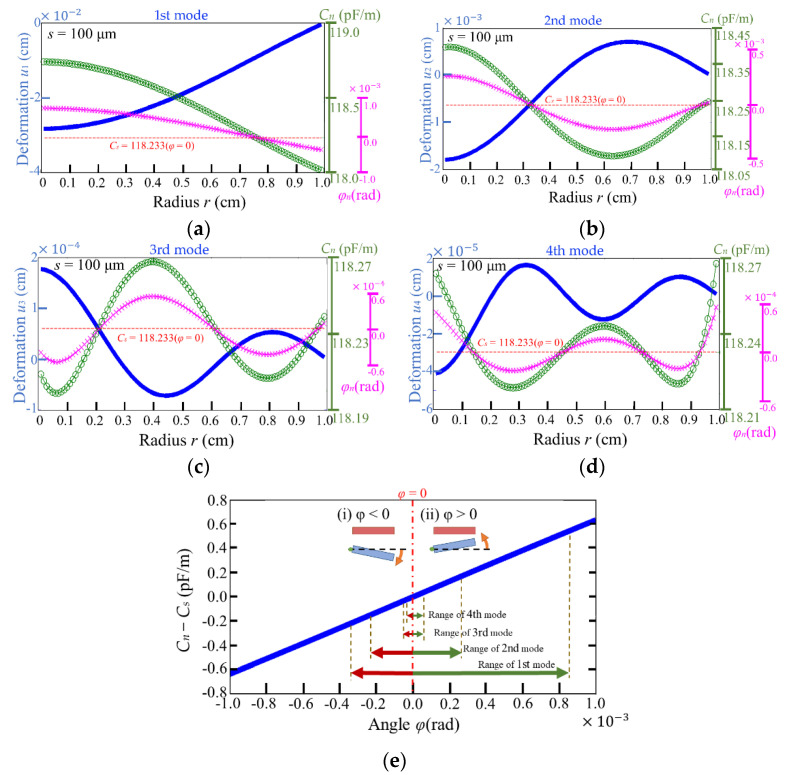
The membranous deformation *u_m_* (blue), the *n*-th angle *φ_n_* (pink) and capacitance (green) per unit longitudinal length *C_n_* (*C_in_* + *C_out_*) along the radius of the membrane in various modes: (**a**) 1st mode, (**b**) 2nd mode, (**c**) 3rd mode, and (**d**) 4th mode. *C_s_* = 118.233 pF/m means the capacitance before vibration. (**e**) The plot of (*C_n_* − *C_s_*) vs. angle of electrode.

We assume that the subsidence of the membranous center point is *δ* = 0.5 *R*, the height of rigid electrode is *h* = *s*/0.075, the radius of the membrane is *R* = 1 cm, and *λ_m_t* = 2π, so the deformation of the membrane in 1st–4th modes, shown in Figure 6, can be obtained from Equation (4). With Equation (5), we have the *N*-capacitive rings on the circular membrane, and each capacitive ring has its own inside angle *φ_n_*, where *n* = 1 to *N* represents the *n*-th capacitive ring. According to Equations (6)–(11) and the curve of the deformed membrane, the *n*-th capacitance per unit longitudinal length *C_n_* can be evaluated.

Figure 6 presents the distributions of the capacitances per unit length *C_n_* (*C_in_*_,*n*_ + *C_out_*_,*n*_) along the radius of the membrane in the 1st–4th modes, and the ranges for the modes are 118.0156–118.7415 pF/m in the 1st mode, 118.0884–118.3985 pF/m in the 2nd mode, 118.1989–118.2679 pF/m in the 3rd mode, and 118.2186–118.2679 pF/m in the 4th mode. It is clear that the range in the lower mode is wide, which reveals that the first mode is beneficial for energy harvesting. The result also shows that the larger angle between electrodes leads to the larger absolute capacitive variation. In other words, the minimum absolute capacitive variations are shown at the angles, which are approximately zero (*φ* = 0°). The 0° angle occurs at the corresponding inflection points in the deformation curves, at which the absolute capacitive variation is zero. Specifically, one point is observed in the 1st mode (*r* = 0.76, Figure 6a), two points are observed in the 2nd mode (*r* = 0.33 and 0.97, Figure 6b), three points are observed in the 3rd mode (*r* = 0.21, 0.61, and 0.96, Figure 6c) and four points are observed in the 4th mode (*r* = 0.14, 0.45, 0.73 and 0.93, Figure 6d). We note that the change in the capacitance is correlated to the angle between electrodes such that one can observe the capacitive variation could be positive or negative along the radius of the membrane (the relationship between capacitive variation and angle is shown in Figure 6e). Despite the existence of negative values that affect the efficiency of conversion of mechanical vibration to electrical energy, overall, the influence of the positive values still overrides that of the negative ones. It should also be pointed out that the negative values can only be obtained from a theoretical analysis, which thus indicates the strength of our approach.

**Figure 7:**
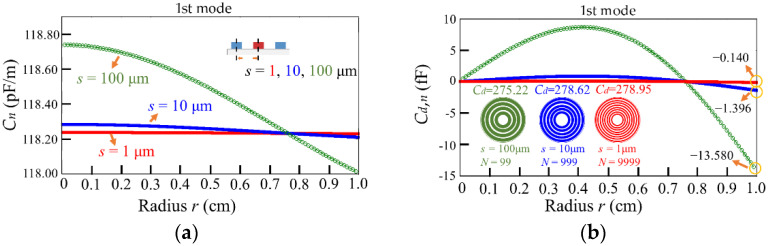
(**a**) The capacitance per unit longitudinal length *C_n_* (*C_in_* + *C_out_*) and (**b**) the capacitive variation in the electrical rings (*C_d_*_,*n*_) along radius of membrane for the 1st mode at the gaps *s* = 100, 10, and 1 μm.

In terms of the size of the capacitive gap, we compare the capacitance of the rings with three types of gaps (*s* = 1, 10, and 100 μm, and the relative rings are 9999, 999, and 99 circles) for the 1st mode. Figure 7a illustrates that the ring with *s* = 100 μm exhibits the highest capacitance per unit longitudinal length due to a larger *φ_n_*. In addition, Figure 7b displays the capacitive variation in the three types of electrical rings around the circle center along the radius, which are obtained from Equation (11). The capacitive variations of the electrical ring are −13.580–8.691, −1.396–0.877, and −0.140–0.088 fF for the membranes with gaps of 100, 10, and 1 μm, respectively. However, in the first mode, the totals of capacitive variations *C_d_* for the three gaps (*s* = 1, 10, and 100 μm) are similar, and their values are indicated in Figure 7b.

**Figure 8:**
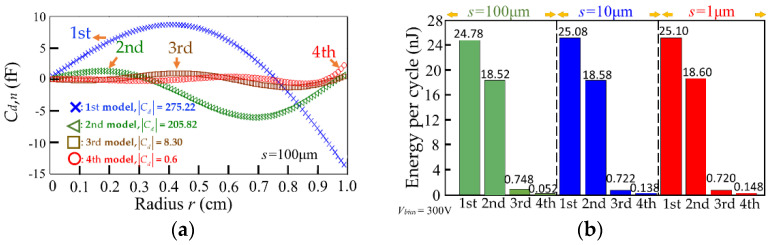
(**a**) The capacitive variation in the rings of *s* = 100 μm along the radius of the membrane (*C_d_*_,*n*_) and (**b**) energy output per cycle for the rings of *s* = 100, 10, and 1 μm when *V_bias_* = 300 V for energy harvesters in the 1st–4th modes.

Concerning the modal shape, we obtain the capacitive variation in the electrical rings along the radius of the membrane in 1st–4th mode with *s* = 100 μm (see Figure 8a). It is found that the amplitude of the *C_d_*_,*n*_ decreases with increasing modes. We also notice that the *C_d_*_,*n*_ can have positive and negative values at any given moment during vibration, as shown in Figure 8a. In addition, with Equation (12), the energy output per cycle for the three types of energy harvester can be calculated. Figure 8b shows the results that the energy outputs for the three types of rings of *s* = 100, 10, 1 μm are similar, and the energy output greatly decreases in the 3rd and 4th modes due to smaller deformation. We see that all of the energy per cycle falls within the range of 0.052–25.10 nJ, which indicates that the energy output is independent of the gap size. As stated above, under each mode, the absolute values of capacitive variation │*C_d_*_,*n*_│, and the angle change *φ_n_* are positively correlated. Since the angle profiles *φ*(*r*) for different gap sizes are the same, which indicates the sums of *φ_n_* for different gap sizes are equal, we can find that the sum of *C_d_*_,*n*_, i.e., *C_d_*, for different gap sizes will be maintained. This accounts for the evaluation in Figure 8b that the energy output of the three devices is numerically close to each other. In short, gap sizes do not affect the energy output in our device.

In order to raise the energy output of the harvester in the first-mode deformation, we consider only the IRE coating between *r* = 0–0.76 of the membrane (see Figure 7b). Thus all of the capacitive variations are positive, and the calculated energy output is raised to 38.68 nJ per cycle. Assuming the vibration is 30 Hz controlled by adjusting *T_p_*, a power density of 0.37 μW /cm^2^ would be achieved for this IRE design on the circular membrane. Table 3 compares the parameters used in our study to assess power densities (IRE) with those used in previous studies (IDE).

In Table 3, the power density of the variable-capacitance harvester is associated with the values of the gap, the voltage, and the frequency. However, the power density of the IRE design is generally smaller than that of the IDE designs. This can be explained by Figure 5, showing that, compared with the non-rotating electrodes, the rotating electrodes give rise to a smaller capacitance difference per vibration cycle under the same normalized displacement. Nevertheless, the IRE device is featured in large gap distance (millimeter-scale), and it can be manufactured by a printed circuit board (PCB) technology at a low price [22]. Comparatively, the bulk of IDE designs is operated by applying the high-frequency vibration source on a small-scale device, which, however, has a higher resonance frequency due to its size [23]. Since the majority of the natural vibration sources are low-frequency within the range of 30–200 Hz [24], our membranous device, which controls the frequency of the first mode at 30 Hz by adjusting the tension force of the membrane and could be triggered with wind, water flow, and sound wave easily, is a more feasible design.

## 4. Conclusions

A mathematical model combining variable capacitance and vibrated circular membrane was proposed to analyze the energy output of capacitive rings on circular membranes in vibration 1st–4th modes. We obtain the capacitive variation in inclined-plate capacitors by algebraic calculation. This study also shows a positive correlation between the energy output and electrodes′ angle. By way of illustration, the first mode performs the best energy output, which is 1.44 times that of the second mode, 33.13 times that of the third mode, and 476.53 times that of the fourth mode. Most non-rotating electrode design models need to minimize the gap size to improve energy output [25]; fortunately, in our design, it is proven that the gap size between the rotating electrodes is independent of the energy output. Therefore, the IRE mechanism has a high potential to be applied in the millimeter-scale and has the advantage of being low-cost in manufacturing.

## Figures and Tables

**Table 1:** Values of *α_m_* and the first-order Bessel functions of the first kind for modes 1–4 [15].

*m*	*α_m_*	*J*_1_(*α_m_*)
1	2.40483	0.51915
2	5.52008	−0.34026
3	8.65373	0.27145
4	11.79153	−0.23246

**Table 2:** The successive coordinate transformations of the inclined plate.

Plane	Used Transfer Equations	Point-A	Point-B	Point-C	Point-D
*z_p_*-plane		*r*_1_e*^i^^φ^*	(*r*_1_ *+ l*_1_)e*^i^^φ^*	*r*_2_	*r*_2_ + *l*_2_
*t_p_*-plane	*t_p_* = *Mz_p_^π^*^/*φ*^ + *M*_0_	(α, 0)	(−1, 0)	(β, 0)	(1, 0)
*ξ_p_*-plane	*ξ_p_* = 0.5 (1 − α)(1 + *t_p_*)/(*t_p_* − α)	(−∞, 0)	(0, 0)	(1/*k*^2^, 0)	(1, 0)
*ζ_p_*-plane	Schwarz-Crystoffel	(0, *iK′*)	(0, 0)	(−*K*, *iK′*)	(−*K*, 0)

**Table 3:** Parameters of various electrostatic VEHs.

Author	Ref	Output Power (μW)	Device Area (cm^2^)	Power Density (μW/cm^2^)	Frequency (Hz)	Gap (μm)	Voltage (V)
This study ^1^	-	1.16	π	0.37	30	100	300
Tsutsumino ^2^	[18]	38	3.00	12.67	20	15	950
Suzuki ^2^	[4]	1	3.05	0.33	63	50	180
Roundy ^2^	[19]	11	1.00	11.00	100	0.25	10
Basset ^2^	[20]	0.061	0.66	0.92	250	1.7	8
Hoffmann ^2^	[21]	3.5	0.30	11.67	1300–1500	2.5	50

^1^ Using the IRE design (pure model). ^2^ Using the IDE design (experimental).
